# Monolithic integration of AlGaInP-based red and InGaN-based green LEDs via adhesive bonding for multicolor emission

**DOI:** 10.1038/s41598-017-11239-4

**Published:** 2017-09-04

**Authors:** Chang-Mo Kang, Seok-Jin Kang, Seung-Hyun Mun, Soo-Young Choi, Jung-Hong Min, Sanghyeon Kim, Jae-Phil Shim, Dong-Seon Lee

**Affiliations:** 1 School of Electrical Engineering and Computer Science, Gwangju Institute of Science and Technology (GIST), 123 Cheomdangwagi-ro, Buk-gu, Gwangju 61005 Korea; 2 Korea Institute of Science and Technology (KIST), 5, Hwarang-ro 14-gil, Seongbuk-gu, Seoul, 02792 Korea

## Abstract

In general, to realize full color, inorganic light-emitting diodes (LEDs) are diced from respective red-green-blue (RGB) wafers consisting of inorganic crystalline semiconductors. Although this conventional method can realize full color, it is limited when applied to microdisplays requiring high resolution. Designing a structure emitting various colors by integrating both AlGaInP-based and InGaN-based LEDs onto one substrate could be a solution to achieve full color with high resolution. Herein, we introduce adhesive bonding and a chemical wet etching process to monolithically integrate two materials with different bandgap energies for green and red light emission. We successfully transferred AlGaInP-based red LED film onto InGaN-based green LEDs without any cracks or void areas and then separated the green and red subpixel LEDs in a lateral direction; the dual color LEDs integrated by the bonding technique were tunable from the green to red color regions (530–630 nm) as intended. In addition, we studied vertically stacked subpixel LEDs by deeply analyzing their light absorption and the interaction between the top and bottom pixels to achieve ultra-high resolution.

## Introduction

Inorganic light-emitting diodes (LEDs) are the brightest, and most efficient and stable light source for displays^1–3^. In the current display industry, inorganic LEDs are mainly used as backlights for thin-film-transistor liquid-crystal displays or red-green-blue (RGB) subpixels for outdoor LED displays. Recently, as the interest in microdisplays such as the ones used in smart phones, smart watches, and head-mounted displays (HMDs) has increased, much effort has been made to apply highly efficient inorganic LEDs as their microdisplay light source^4–10^. However, technical issues still remain when using inorganic LEDs as the direct light source (self-radiative light source) for RGB full color microdisplays.

The color of the light emitted from an inorganic LED is determined by the bandgap energy of the material in the active region. Theoretically, the bandgap energy of InGaN can be tuned from the infrared (0.69 eV) to ultraviolet (3.4 eV) by varying the composition of In in an InGaN alloy^11, 12^. However, the internal and external quantum efficiencies of InGaN-based LEDs decrease so abruptly toward the red spectral region that InGaN-based materials are only used for green and blue light emission sources^13, 14^. Therefore, it is essential to combine InGaN with highly efficient red light emission materials such as AlGaInP for RGB full color displays^15^. Conventionally RGB LEDs are separately diced from each InGaN- or AlGaInP-based wafer and individually arranged on display panels to form RGB pixels via robotic manipulation. However, robotic arms limit the scaling to a few tens of micrometers for the LED chip size due to the difficulty in picking and placing the large number of tiny RGB LED pixels, which is not a suitable method to realize high resolution (HR) microdisplays.

Recently, many studies have been conducted to achieve HR microdisplays by accurately and effectively arranging each red, green, and blue LED to achieve a chip size of under a few tens of micrometers. Some research groups have demonstrated both passive-matrix and active-matrix LED microdisplays by transferring 10 μm pixel films with an elastomer stamp^10, 16, 17^. Other research groups have proposed methods for transferring many-pixel films by using elements to control the electrostatic or electromagnetic properties to pick and place the pixel films^18, 19^. Although the scaling issues have been overcome by introducing either stamps, or electrostatic or electromagnetic methods, transfer yield problems such as missing pixels on a display panel during the LED transfer process still remain. In addition, these approaches sometimes require the high-cost laser lift-off (LLO) process to remove the substrate of epitaxial layer^16, 20, 21^.

Therefore, alternative integration methods without the use of a transfer machine would be beneficial to realize high-resolution RGB full color displays in an industrial setting. One route could be achieved through interface designs such as ZnO/GaN or growth techniques of hetero junctions like n-p-n structures, although the lighting efficiency needs to be improved^22, 23^. Another group suggested stacking wafer-level thin films of the III-V-based LEDs to realize multicolor pixels^24^.

Herein, we integrated two materials having different bandgaps via adhesive bonding and a chemical wet etching process. The InGaN- and the AlGaInP-based LEDs were monolithically integrated for green and red emission, respectively. Our main strategy was to transfer the entire red LED epitaxial layer onto a green LED wafer and then divide the pixels via fabrication. Unlike conventional methods, we were able to minimize the pixel size to several microns because our method only depended on lithography resolution. Furthermore, it required transferring the entire LED epitaxial layer only once with a much improved transfer yield compared to existing methods in which each RGB LED is transferred individually and repeatedly. The other advantage of our approach is a dramatic cost reduction through ruling out complex fabrication steps such as the LLO process and does not require the use of pick-up/transfer machines. Furthermore, we were able to realize not only the laterally arranged subpixel (LAS)-type structure but also the vertically stacked subpixel (VSS)-type array structure which increases the display resolution by up to three times more than the LAS-type^24, 25^.

We fabricated two types of monolithic integrated red on green LEDs by using a bonding technique for both the LAS-type, and the VSS-type array structures. For a LAS-type structure, we specifically analyzed the optical and electrical properties of the integrated red and green subpixels (after adhesive bonding) by comparing reference samples (before bonding). We were able to realize multicolor emission from the green to red wavelengths by controlling the input power of each green and red subpixel. Furthermore, we intensively studied the light absorption and photoluminescence (PL) effect for the VSS-type structure to minimize the light interference effect and obtain the pure color of the light emission.

## Results and Discussion

**Figure 1:**
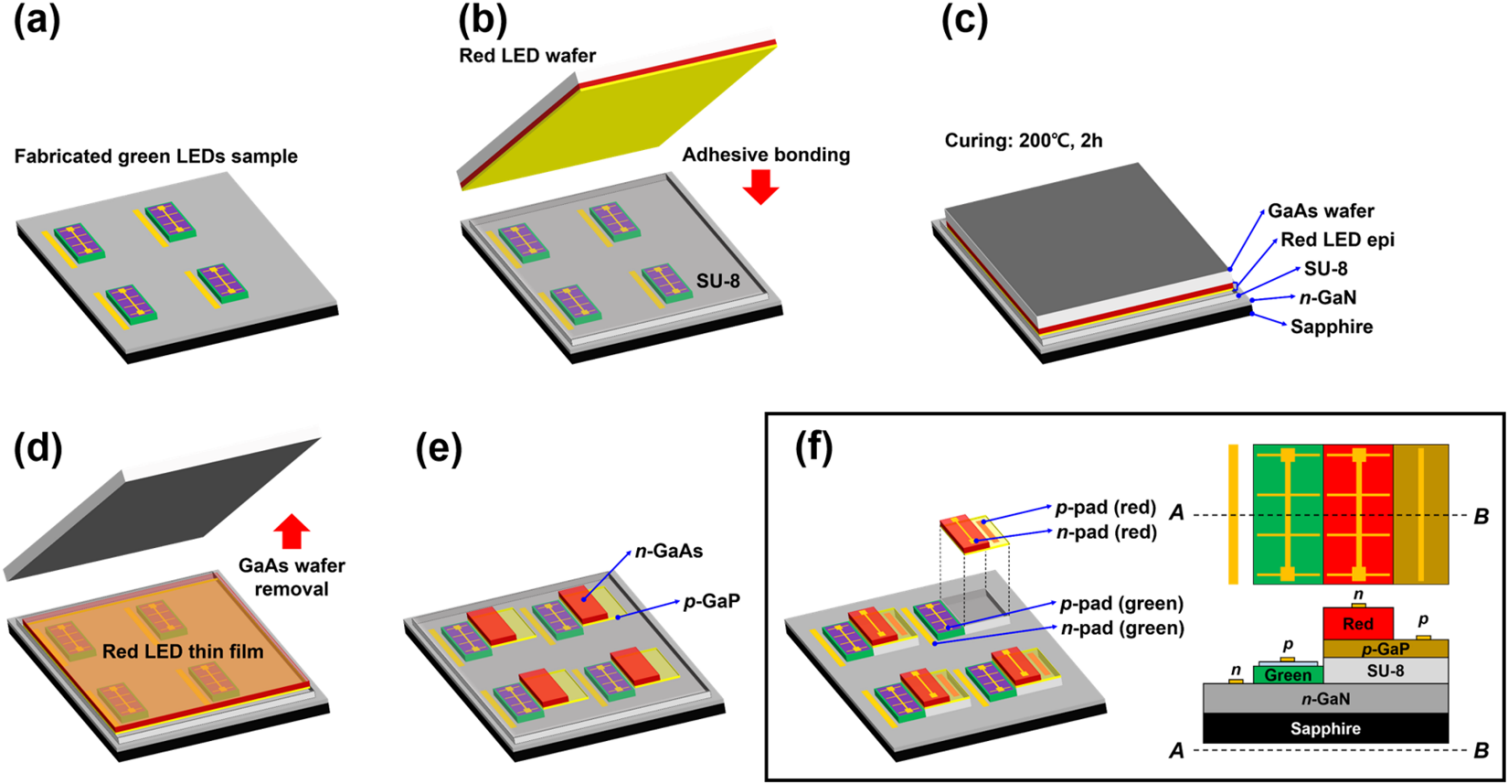
Schematic fabrication process of dual color LEDs: (**a)** fabrication of InGaN green LEDs, (**b)** adhesive bonding of a bare red LED wafer and fabricated green LEDs, (**c)** curing of the adhesive material inserted between the bare red LED wafer and the fabricated green LEDs, (**d)** GaAs substrate removal by chemical wet etching, (**e)** mesa pattern of the red LED thin film, and (**f)** formation of the *n*- and *p*- electrodes of the red LEDs.

Figure 1 shows a schematic of the fabrication process of the dual color LEDs realized using the proposed adhesive bonding and chemical wet etching approach (see the method section for details). In this experiment, the InGaN-based green LED epitaxial structure grown on a double-sided polished sapphire (DSPS) substrate and the AlGaInP-based red LED epitaxial structure grown on a GaAs substrate were used for green (~530 nm) and red (~630 nm) light emission, respectively (see Supplementary Fig. S1). We used the bonding process after the fabrication of the green LEDs. The key point is that we transferred the red LED epitaxial layer onto the green LEDs using bonding and chemical wet etching and then made the red LED subpixels. The size of the green and the red subpixels was 1 × 0.5 mm^2^.

**Figure 2:**
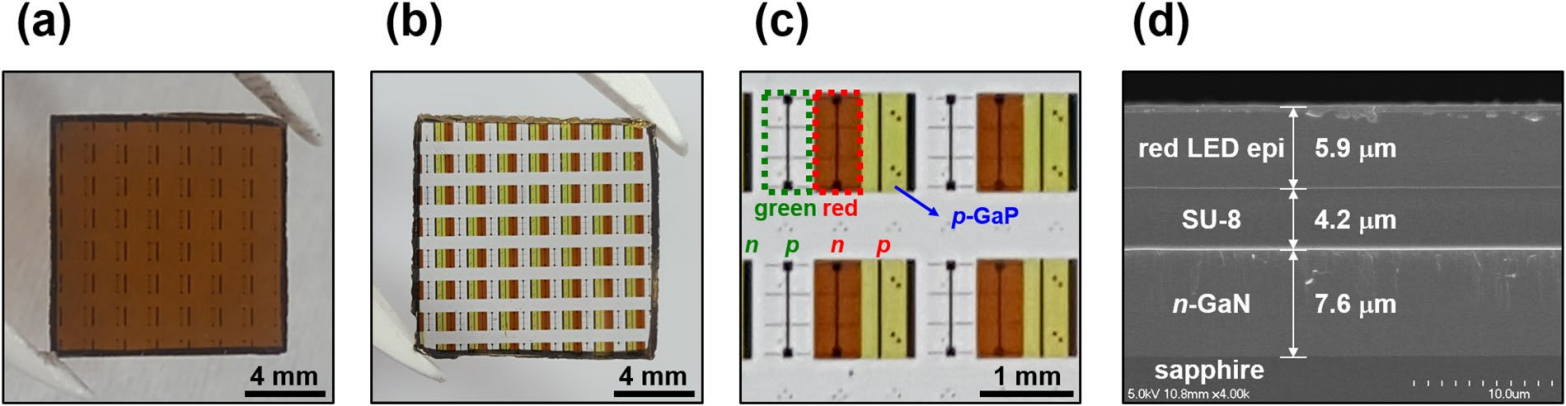
Bonding reliability of the device fabricated by adhesive bonding and chemical wet etching: (**a)** photograph of the AlGaInP-based red LED thin film transferred completely onto the green LED epilayer (the transferred film does not show any cracks or winkles); (**b)** a photograph of the final device with both green and red subpixels arranged laterally; (**c)** a magnified photograph of the final device; and (**d)** a cross-sectional scanning electron microscopy (SEM) image of the red LED thin film bonded onto the *n*-GaN epitaxial layer by SU-8 without any cracks or air voids.

The photograph of the sample in Fig. 2(a) shows that the AlGaInP-based red LED thin film was completely transferred onto the green LED sample (1.2 × 1.2 cm^2^) by removing the GaAs substrate via chemical wet etching, and the transferred film had no cracks or wrinkle. The key point of adhesive bonding was to form an interface completely free from air voids by controlling the curing temperature. Moreover, temperature was also considered in other fabrication processes such as annealing for ohmic contact. Thus, we optimized both the curing and final annealing temperatures so that wrinkles and cracks did not occur, and in this experiment, these were set to 200 °C and 250 °C, respectively (see Supplementary Fig. S2). Figure 2(b,c) show the final monolithic device in which red LED subpixels were laterally arranged at the side of the green LED subpixels; all of the subpixels were clearly preserved and there were no missing pixel positions since the red LED subpixels were fabricated after only one transfer of red LED epitaxial film. The interface between the red and green LED epitaxial layers bonded by SU-8 adhesive material is shown in the SEM image in Fig. 2(d); the thickness of the SU-8 was about 4.2 μm, and the SU-8 was sandwiched uniformly between the red and green LED layers without any air voids or defects. These results are very meaningful in that the integration of the AlGaInP-based and InGaN-based LEDs was realizable by the adhesive bonding and a standard semiconductor process without using the costly LLO process.

**Figure 3:**
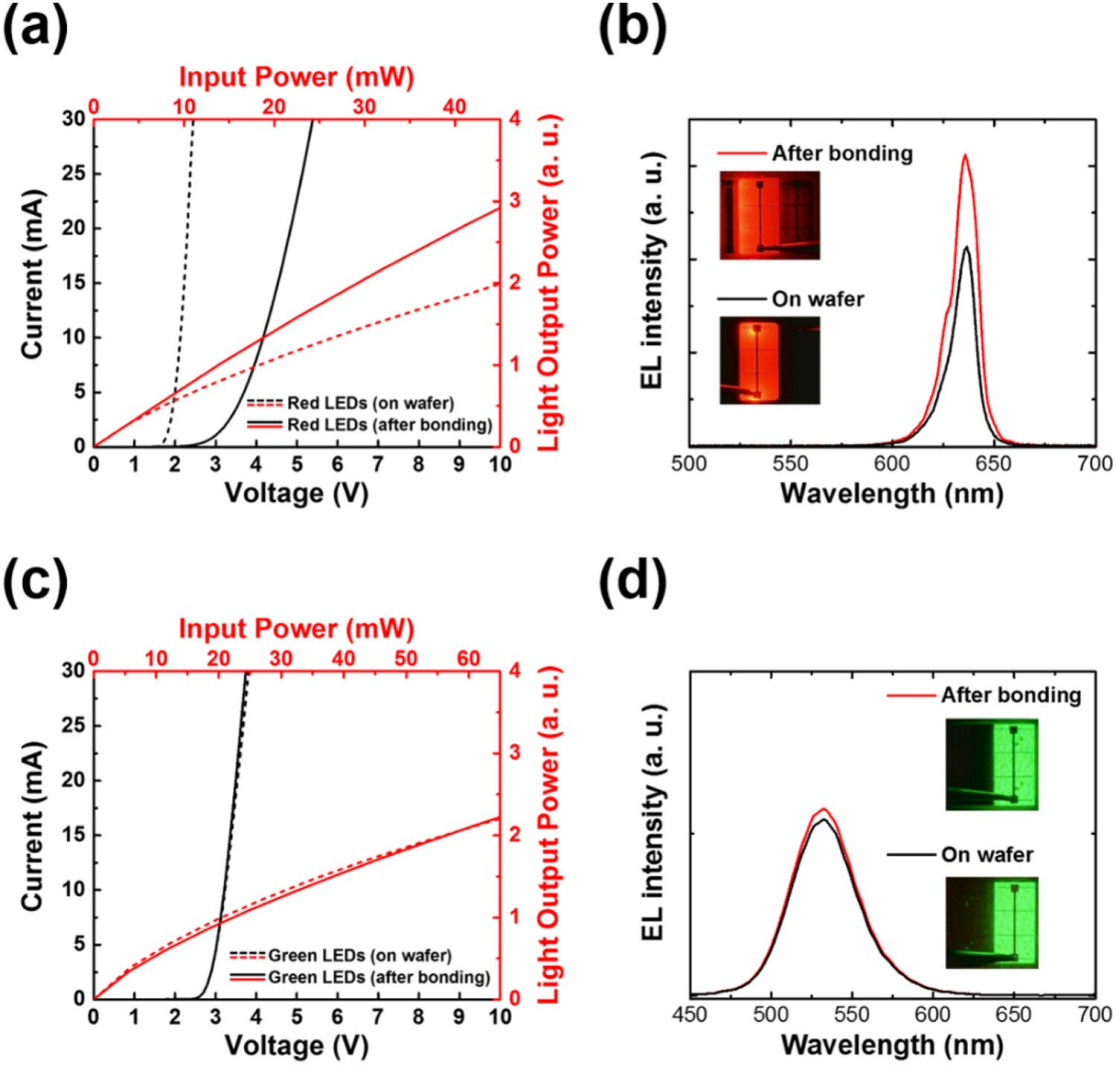
Electrical and optical properties of the transferred red and green LED subpixels: (**a)** current-voltage (I-V) and electrical input power-light output power (EIP-LOP) characteristics and (**b)** electroluminescence (EL) spectra and microscope images of the transferred red LEDs and those on a wafer, and (**c)** I-V and EIP-LOP characteristics and (**d)** EL spectra and microscope images of the green LEDs before and after bonding. All samples were operated at an EIP of 20 mW.

Figure 3(a) presents the current-voltage (I-V) and electrical input power-light output power (EIP-LOP) characteristics of the transferred red LEDs (after bonding). For the comparison, we fabricated red LEDs without bonding and chemical wet etching (on a wafer) as a reference. As shown by the I-V data, electrical degradation occurred in the transferred red LEDs since their turn on voltage was measured as 4.8 V (@20 mA), which is 2.5 V higher than those on the wafer. The main reason for the degradation in electrical properties is that the *p*-type metal contacts of the transferred red LEDs were formed in a low *p*-doping region compared to the red LEDs on the wafer, which was mainly caused by formation of an inverted LED structure during the bonding process. In the case of the red LEDs on the wafer, *p*-type metal contacts were formed on the *p*-GaP surface, while those of the transferred red LEDs were formed on the inner *p*-GaP region, which is located at a depth of 2 μm from the surface of the wafer. The dopant material and the doping concentration differed by the depth of the *p*-GaP layer (see Supplementary Fig. S3). The doping concentrations of the top *p*-GaP(C) and the inner *p*-GaP(Mg) were at the 10^19^ level (high) and 10^18^ level (low), respectively.

Despite of the degradation in the electrical properties of the transferred red LEDs, there was significant optical improvement compared to the red LEDs on the wafer, as shown in the EIP-LOP characteristics in Fig. 3(a). The EL spectra and the microscope images in Fig. 3(b) demonstrate the optical improvement of the transferred red LEDs and it was attributed to the removal of the GaAs substrate which absorbs a large amount of red light. Therefore, the transferred red LEDs showed ~30% higher EL intensity than the red LEDs on the wafer at the same EIP (20 mW). In addition, a shoulder peak at ~625 nm, which was hardly observable in the EL spectrum of the red LEDs on the wafer, was clearly shown in the EL spectrum of the transferred red LEDs. We believe that the hidden shoulder peak, which was absorbed by the GaAs substrate before removal of the substrate, appears to have become strong as the GaAs substrate was removed (see Supplementary Fig. S4). The effect of absorption by the GaAs substrate is also shown in the microscope images. The red LEDs on the wafer had no light emitted from the side of the pixels and looked very black except for the pixels themselves. On the other hand, the transferred red LEDs emitted light from their sides.

Figure 3(c,d) shows the I-V, EIP-LOP and EL spectrum (@ 20 mW) for green LEDs before and after bonding; their turn on voltage, series resistance, and peak wavelength after bonding were 3.4 V (@20 mA), 23 Ω, and 530 nm, respectively, which were the same as those before bonding. In addition, the optical power after and before bonding was also similar. This infers that the optical and the electrical properties of the green LEDs were almost unaltered by the bonding process because the heat treatment after bonding was implemented at a lower temperature than the fabrication temperature of the green LEDs. Consequently, both the red and green LEDs, even after bonding, show good device performance owing to reliable fabrication and they were sufficiently usable for microdisplay application.

Additive color mixing of two or more light sources is used in many applications, and particularly in displays, the color of a certain pixel on a screen is determined by the mixing of red, green, and blue light sources. The precise color production of the pixel is a very important factor in the evaluation of display performance, and for this reason, the fabricated dual color LEDs needed to be evaluated for color tuning capability. In order to verify that the color mixing of the dual color LEDs fabricated by the bonding technique can be controlled as intended, we measured the CIE color coordinates and EL spectra by seven different modes using four probes. The seven modes were divided by the different optical power ratios of each light source as follows: [(Green: Red)] = [(6:0), (5:1), (4:2), (3:3), (2:4), (1:5), (0:6)]. In the test, the total optical powers of the dual color LEDs emitted for the seven modes were set to analyze color coordinates. We adjusted the electrical input power of the red and green LEDs to match each optical power ratio for the seven modes as intended.

**Figure 4:**
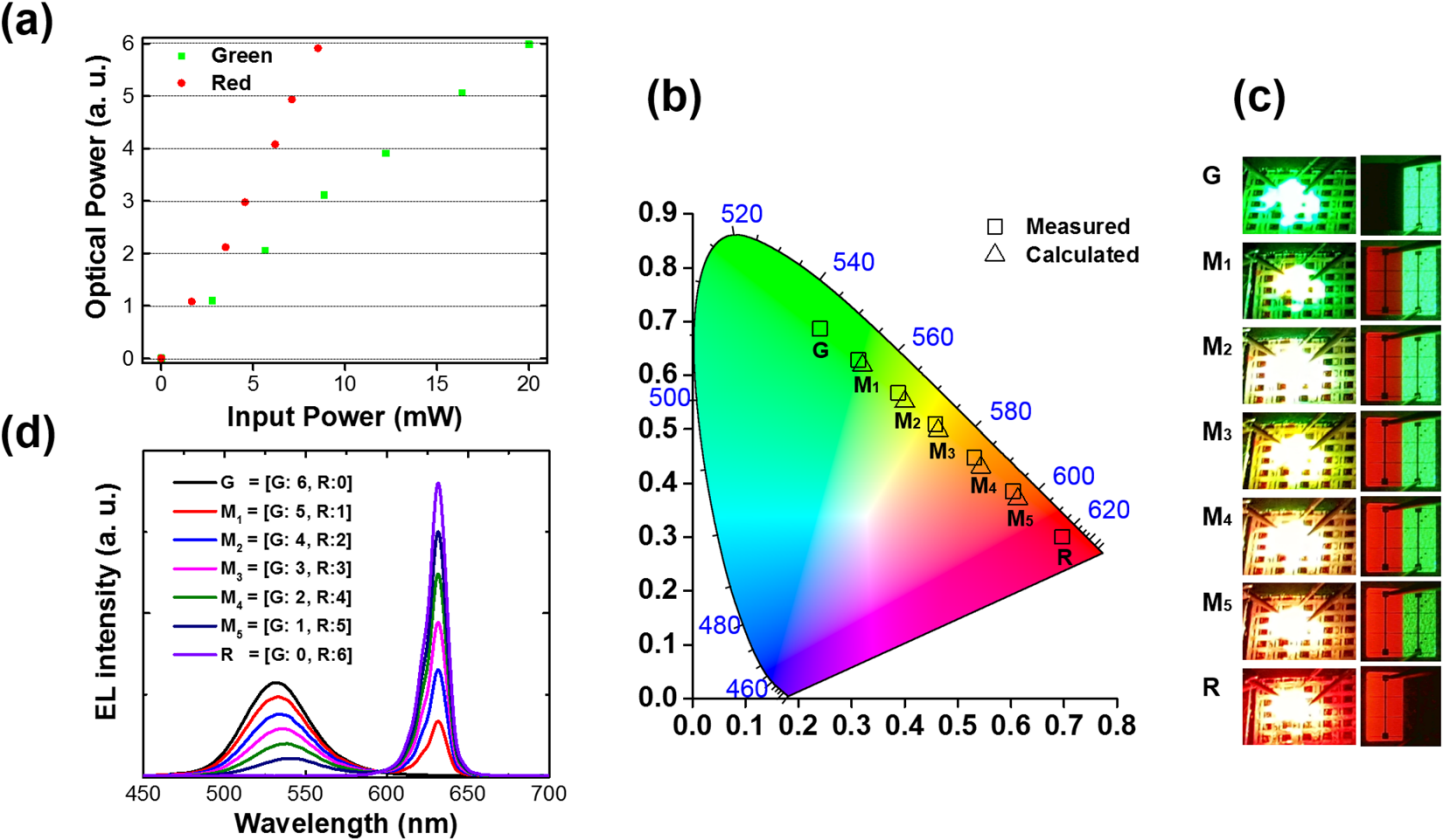
Performance of additive color mixing on the dual color LEDs having a LAS-type array structure realized by adhesive bonding: (**a**) optical output and input powers tuned in the green and red LED subpixels, and (**b**) CIE color coordinates, (**c**) photographs (left) and microscope images (right), and d) EL spectra of the dual color LEDs for the seven color modes. For reference, the CIE coordinates for the seven color modes were calculated using equation (3).

Figure 4(a) presents the plots of the input and the optical powers of the two light sources for the seven modes. Each optical power setting for the green and the red LEDs was divided into seven gray levels according to input power, and each combination of optical power of the two LED types determined their color. The color coordinates of the mixed lights operating for the seven modes can be calculated according to the following equations:

1 $${L}_{1}=\overline{x}({\lambda }_{1}){P}_{1}+\overline{y}({\lambda }_{1}){P}_{1}+\overline{z}({\lambda }_{1}){P}_{1},$$

2 $${L}_{2}=\overline{x}({\lambda }_{2}){P}_{2}+\overline{y}({\lambda }_{2}){P}_{2}+\overline{z}({\lambda }_{2}){P}_{2},$$

3 $$x=\frac{{x}_{1}{L}_{1}+{x}_{2}{L}_{2}}{{L}_{1}+{L}_{2}},\,\,\,\,\,y=\frac{{y}_{1}{L}_{1}+{y}_{2}{L}_{2}}{{L}_{1}+{L}_{2}},$$

where [$\overline{x}(\lambda )$, $\overline{y}(\lambda )$, $\overline{z}(\lambda )$], [P_1_, P_2_], and [x_1or2_, y_1or2_] are the color matching functions, the optical powers and color coordinates of the two light sources, respectively.

Table 1 shows the color coordinates measured for the seven modes and those calculated using equation (3). By comparing these two values, we can confirm that the experimental results were almost the same as the calculated values. In addition, as shown in Fig. 4(b), each color coordinate of the mixed colors is located on a straight line connecting the color coordinates of the two light sources into approximately six equal parts. This means that we were able to produce the precise color of the pixel by the optical power ratio. Figure 4(c) shows a series of pictures of the LEDs corresponding to the color coordinates in Fig. 4(b). To the left and right of Fig. 4(c) are photographs taken at far-field and magnified microscope images, respectively. From the magnified microscope images, we can confirm that we were able to independently operate the red subpixel on the left and the green subpixel on the right. Although the color of the mixed light cannot be recognized from the magnified image due to separate emissions, we were able to recognize the color of the mixed light in the image in the far field. As shown in these images on the left of Fig. 4(c), the colors of the mixed light emitted for the seven modes were consistent with those of the color coordinates in Fig. 4(b). Figure 4(d) shows the EL spectra consisting of the superposition of each EL spectrum of the green and the red LEDs measured for the seven modes. The spectra of the single color modes had only one peak wavelength of either 530 or 630 nm, while those of the mixed color modes (such as M1-M5) have dual peak wavelengths of both 530 and 630 nm. From these results, it is evident that the dual LEDs fabricated by the bonding technique are applicable as subpixels for self-radiative light source by tuning the color from green to red.

**Table 1:** Calculated and measured CIE color coordinates of the dual color LEDs having a LAS-type array structure for seven color modes.

Optical Power Ratio	G	M_1_	M_2_	M_3_	M_4_	M_5_	R
	G:6, R:0	G:5, R:1	G:4, R:2	G:3, R:3	G:2, R:4	G:1, R:5	G:0, R:6
Calculated	(0.24, 0.69)	(0.32, 0.62)	(0.40, 0.55)	(0.46, 0.50)	(0.54, 0.43)	(0.61, 0.37)	(0.69, 0.3)
Measured	(0.24, 0.69)	(0.31, 0.63)	(0.39, 0.57)	(0.46, 0.51)	(0.53, 0.45)	(0.60, 0.39)	(0.69, 0.3)

**Figure 5:**
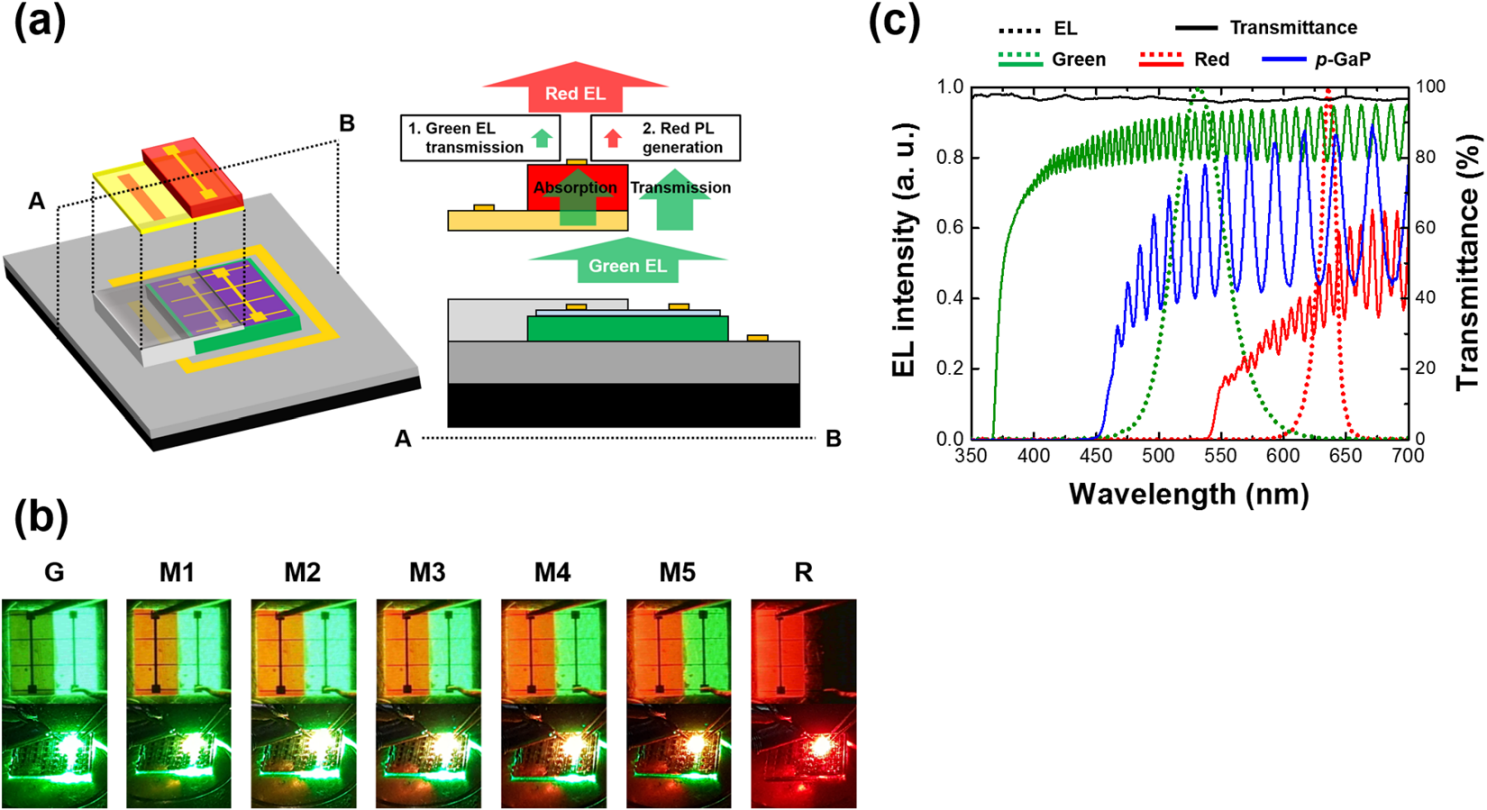
Dual color LEDs in a VSS-type array structure: (**a)** Schematic image; (**b)** photographs (lower) and microscope images (upper) for the seven modes; and (**c)** solid lines showing transmittance of the red LED thin film (red), *p*-GaP layer (blue), and green (green) LED layer on DSPS, and dotted lines showing the EL spectra of the transferred red (red) and the underlying green (green) LEDs.

Although the LAS-type array structure is commonly used for full color displays, the VSS-type array structure is more promising to achieve ultra-HR in the next-generation of displays. If we optimally stack the red, green, and blue LEDs, the density of integration increases by up to three times more than for the LAS-type structure, resulting in the capability to downsize devices and retain HR. Therefore, we also verified the possibility of a VSS-type structure for the AlGaInP- and InGaN-based dual color LEDs via adhesive bonding, as shown in Fig. 5(a,b) (also see Supplementary Fig. S5). The most important point is that our film transfer method is co-applicable to the VSS-type structure in that it does not need additional pixel arrangement in a vertical direction. Similar to the fabrication process of the LAS-type array structure, the AlGaInP-based red LED epitaxial layer was transferred and then the red subpixels were patterned; all pixels clearly remained without any being damaged or missing.

Although the VSS-type array structure is promising for HR microdisplays, there are many issues to consider. First, the light emitted from the green LEDs was able to be absorbed by the red LED thin film because the bandgap energy of GaP (2.32 eV) and AlGaInP (1.98 eV) for red emission was lower than green (2.36 eV) light. Figure 5(b) shows a series of emission images of a VSS-type device for the seven modes. Here, the left subpixel is a VSS-type. In green mode, the left subpixel (a red LED in the green LED region) emitted a very weak green light compared to the right subpixel. Furthermore, the left subpixels from M1 to M5 show mixed color of red and green, but red light emission is more dominant, which is due to the light emitted by the underlying green LEDs being almost completely absorbed by the red LED thin film.

In order to clarify the green light loss in the VSS-type structure (subpixels of red LED in the green LED region), we measured the transmittance of three samples for which either i) the green LED epitaxial structure was grown on DSPS, ii) the *p*-GaP film was transferred onto DSPS, or iii) the red LED film was transferred onto DSPS (Fig. 5(c)). The transferred red LED film completely absorbed light below ~540 nm and made it possible to transmit light from 540 to 650 nm at approximately 25%. That is to say, only a very small amount of light emitted from the underlying green LEDs was able to penetrate the red LED thin film and a significant amount of green light was absorbed by the red LED thin film. Furthermore, we confirmed that most of the EL from the green LEDs was absorbed by the multiple quantum wells (MQWs) of the red LEDs rather than the *p*-GaP layer, as shown in the transmittances of the *p*-GaP and the red LED thin film. Therefore, it is very important to sequentially stack each subpixel LED by considering its bandgap energy. An LED having larger bandgap energy has to be positioned on one with smaller bandgap energy for multicolor generation in a VSS bonded structure. In our structure, such a problem can be solved satisfactorily by adopting back-light emission toward the DSPS such as a flip-chip structure because the light emitted by the red LEDs was able to be transmitted onto the green LEDs on the DSPS by approximately 85% (Fig. 5(c)).

**Figure 6:**
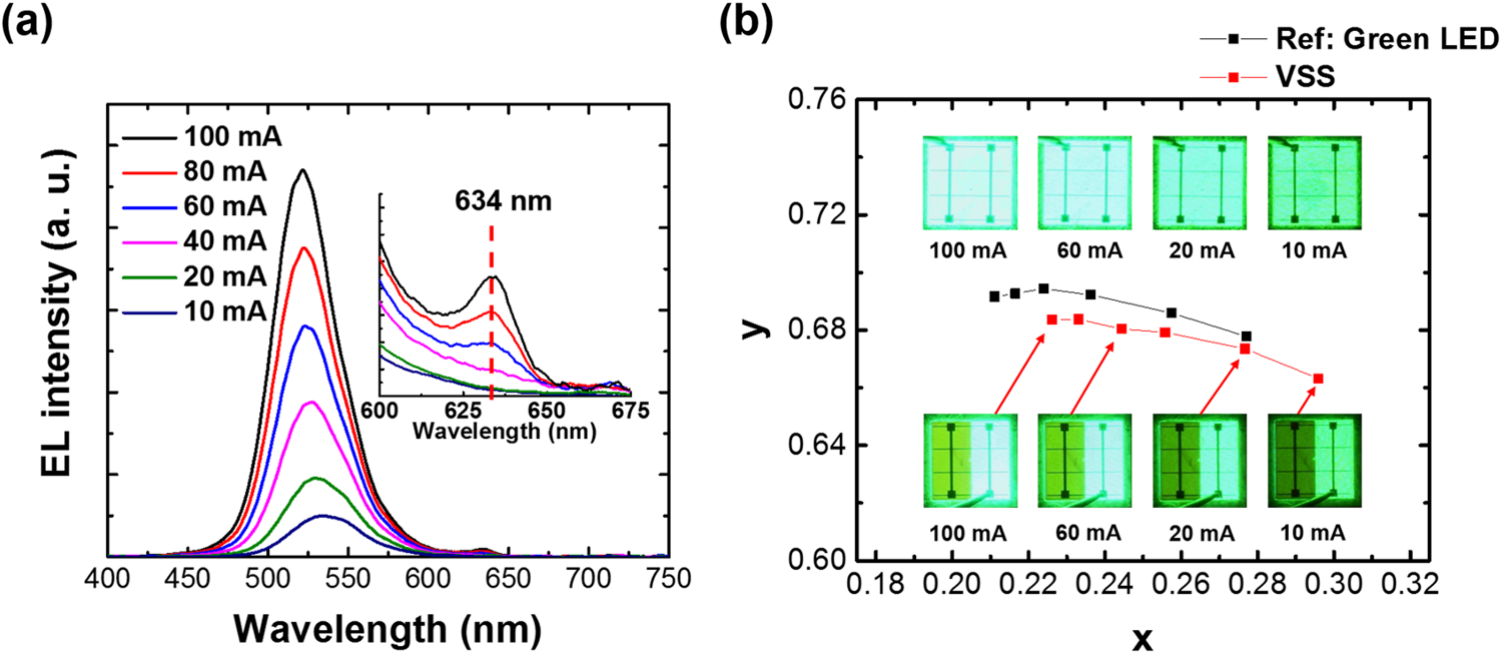
PL phenomena of the red LEDs generated by light emission from the green LEDs: (**a)** EL spectra for different current levels delivered to the green LEDs (the inset EL spectra were magnified to be able to clearly observe the weak PL peak generated by light emission from the green LEDs); and (**b)** CIE color coordinates corresponding to the EL spectra in Fig. 6(a). For reference, the color coordinates of the green LEDs before bonding were also measured.

Another issue was that the light emission of one subpixel LED was able to generate photoluminescence (PL) in another subpixel LED, resulting in color modulation. We applied current levels from 10 to 100 mA to the green LED subpixels only and measured their EL spectra to accurately observe the PL phenomenon at the upper-located red subpixel LEDs, as shown in Fig. 6(a). The EL spectra showed a main peak at ~530 nm for green light emission; however, a minor subpeak at ~ 634 nm corresponding to red light emission appeared at a current level of 60 mA and above. The main peak was blue-shifted from 540 to 530 nm as the current increased from 10 to 100 mA, which was attributed to the band filling effects as reported in many previous studies^26, 27^. However, the minor subpeak at 634 nm was not changed by current variation. Specifically, this means that the green light source from the underlying LEDs affected the upper red LEDs, which then generated the PL.

We quantified the light emission with the PL effect based on EL intensity. Table 2 shows the peak intensity ratio and area ratio of the two wavelength band, which could divide into the green region (450–600 nm) and red region (600–675 nm). It can be seen that the PL component of the red LEDs affected approximately 1.5% of the total EL spectra. To consider how much the PL component affected the color of the pixels, the CIE color coordinates were also measured (Fig. 6(b)). For a fair comparison, the green VSS-type LEDs were compared to those on a wafer (before bonding), in which both color coordinates showed that the x components decreased and the y components increased. In addition, the x and y variations of the VSS and reference samples were almost the same, which means that the blue-shift effect of the green LEDs was much greater than the PL effect generated in the red LEDs. Moreover, since the x and y variations of the two samples were almost the same, we showed that the PL component had little effect on the color change in spite of the high current level. Although the PL effect on unintentional color modulation was small, insertion of a distributed Bragg reflector (DBR) structure in the epitaxial layer or adhesive bonding with a new material containing a DBR structure might optimally exclude the PL effect and unintentional color modulation^28–30^.

**Table 2:** Peak intensity and area ratios calculated in the green (450–600 nm) and the red (600–675 nm) spectral regions from the EL spectra in Fig. 6(a) for current levels above 60 mA.

Current	60 mA		80 mA		100 mA	
Spectrum Region	450–600 nm	600–675 nm	450–600 nm	600–675 nm	450–600 nm	600–675 nm
Peak Intensity	98.64%	1.36%	98.36%	1.64%	98.14%	1.86%
Area	98.6%	1.4%	98.6%	1.4%	98.6%	1.4%

## Conclusion

In this study, we realized the integration of AlGaInP red and InGaN green LEDs using a film transfer method followed by adhesive bonding and a chemical wet etching process. The transferred red LED thin film was completely bonded onto the green LEDs without any cracks or voids, and so all of the red and green LED subpixels showed excellent optical and electrical properties. Furthermore, they were perfectly controllable, which enabled multicolor emission from the green to red wavelength regions. Interestingly, the adhesive bonding and chemical wet etching strategy was suitable for both VSS and LAS structures. From these results, we believe that multicolor integration via our bonding technique is very promising for various small display applications requiring HR, such as smart phones, smart watches, and HMDs.

## Method

### InGaN-based green LEDs

To form the mesa pattern of the green LEDs and expose the *n*-GaN layer for *n*-type electrode formation, a standard photolithography process and inductively coupled plasma (ICP) etching were implemented. Following this, an indium tin oxide (ITO) layer was deposited onto the *p*-GaN layer for current spreading. After deposition of the ITO layer, the sample was annealed at 600 °C for 5 min via rapid thermal annealing. Finally, the Cr/Au (30/300 nm) layers were evaporated onto the ITO layers and *n*-GaN layer for formation of the *n*-type and the *p*-type electrodes using an e-beam evaporator.

### Wafer bonding and GaAs substrate removal

To improve the surface adhesion of the fabricated green and red LED wafers, hexamethyldisilazane was coated onto them. Subsequently, the SU-8 bonding material was applied to the fabricated green LED wafer, and the red LED wafer was attached to the green LED wafer along the direction in which the GaAs substrate was exposed. The sample was placed in a vacuum at room temperature for approximately 40 minutes in order to completely remove air voids in the bonding material. Next, the sample was annealed at 200 °C for 2 h under atmospheric pressure using a conduction oven to cure the bonding material. After this, the GaAs substrate was removed using a NH_4_OH-based solution.

### AlGaInP-based red LEDs

To define all of the subpixels of the red LEDs and to expose the *p*-GaP for *p*-type electrode formation for the red LEDs, a standard photolithography process and wet etching process were implemented. Following this, the *p*-GaP layer was patterned by ICP etching in order to independently isolate the *p*-GaP layer for each red subpixel. For the formation of *n*- and *p*-type electrodes, the Pd/Ge/Au metal layer was evaporated onto the *n*-GaAs and the AuBe/Au metal layer was evaporated on the *p*-GaP layer using the e-beam evaporator, respectively. The sample was annealed at 250 °C for 1 h to improve the electrical properties of the *p*- and *n*-type electrodes. Finally, the SU-8 film was removed by reactive-ion etching in order to expose the *n*- and *p*-type electrodes of the green LEDs.

## Electronic supplementary material

Supplementary Information
