# Supplementary material for: Monolithic integration of AlGaInP-based red and InGaN-based green LEDs via adhesive bonding for multicolor emission
Source: Sci Rep. 2017 Sep 4;7:10333. doi: 10.1038/s41598-017-11239-4 (PMC5583240; doi:10.1038/s41598-017-11239-4)
Supplement: Supplementary file 1 — Supplementary Information [file 41598_2017_11239_MOESM1_ESM.pdf]

Monolithic integration of AlGaInP-based red and InGaN-based green LEDs via adhesive bonding for multicolor emission

Chang-Mo Kang[1], Seok-Jin Kang[1], Seung-Hyun Mun[1], Soo-Young Choi[1], Jung-Hong Min[1], Sanghyeon Kim[2], Jae-Phil Shim[2*], and Dong-Seon Lee[1*]

[1] School of Electrical Engineering and Computer Science, Gwangju Institute of Science and Technology (GIST), 123 Cheomdangwagi-ro, Buk-gu, Gwangju 61005, Korea

[2] Korea Institute of Science and Technology (KIST), 5, Hwarang-ro 14-gil, Seongbuk-gu, Seoul, 02792, Korea

*[1]*E-mail: dslee66 @gist.ac.kr*

*[2]*E-mail: d15509@kist.re.kr*

**These authors contributed equally to this work*

Supplementary Materials

1. The red and green LED epitaxial structures used in this study

2. Optimization of the curing and final annealing temperatures for the complete bonding process

3. Analysis of the electrical degradation of the transferred red LEDs from secondary ion mass spectrometry (SIMS) data

4. Detailed observation of the shoulder peak of the transferred red LEDs and the red LEDs on a wafer

5. Performance verification of the dual color LEDs having a VSS structure

1. Red and green LED epitaxial structure used in this study

Figure S1 (a) and (b) show the AlGaInP-based red and the InGaN-based green LED epitaxial structures used in this study. The red LED structure was grown on a GaAs substrate by metalorganic chemical vapor deposition (MOCVD). A buffer layer (GaAs) was grown on the GaAs substrate and then a GaInP etching stop layer was grown for the GaAs substrate removal. The red LED epitaxial structure consisted of a *n*-GaAs ohmic contact layer, a *n*-GaInP layer, a *n*-AlGaInP, a *n*-AlInP cladding layer, the AlGaInP MQWs, a *p*-AlInP cladding layer, a *p*-AlGaInP tensile strain barrier reducing (TSBR) layer and a *p*-GaP window layer. The green LED structure was grown on the DSPS substrate by MOCVD. Firstly, a *u*-GaN buffer layer was grown on the DSPS substrate, then a *n*-GaN ohmic contact layer, the InGaN/GaN MQWs, and a *p*-GaP ohmic contact layer were grown on the *u*-GaN buffer layer.

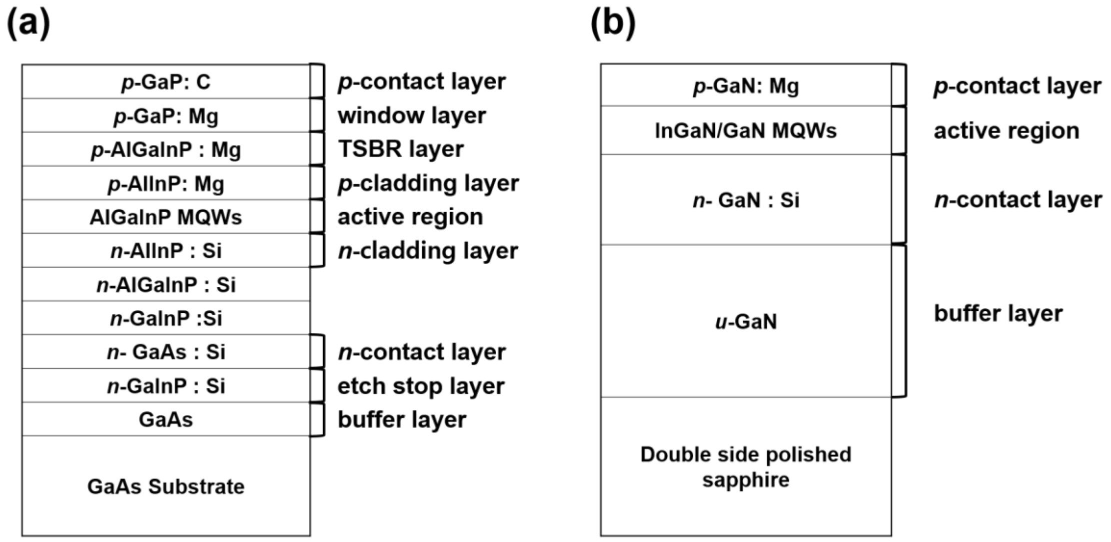

Figure S1. Illustrations of (a) the AlGaInP-based red and (b) InGaN-based green LED epitaxial structures.

2. Optimization of the curing and final annealing temperatures for the complete bonding process

These experiments were carried out to find the optimized curing and final temperatures and to confirm the thermal durability of the transferred LEDs. The SU-8 used as a bonding material is known to have 5% thermal decomposition by weight at 315°C. Figure S2 (a) and (b) show representative samples fabricated at optimized and non-optimized temperatures, respectively. The red LED film was optimally transferred without any cracks at the optimized curing temperature, but the film peeled off or became wrinkled above it. Furthermore, we also checked whether the cured and transferred red LED film was well maintained at the high temperature required for the next fabrication steps (Table S1). The red LED film was completely transferred at a curing temperature range of 160 to 200°C. In addition, the red LED film was able to withstand temperatures up to ~50°C above the curing temperature. Among these conditions, we selected the maximum curing temperature (200°C) and the final bake temperature (250°C) for the adhesive bonding and red LED film transfer.

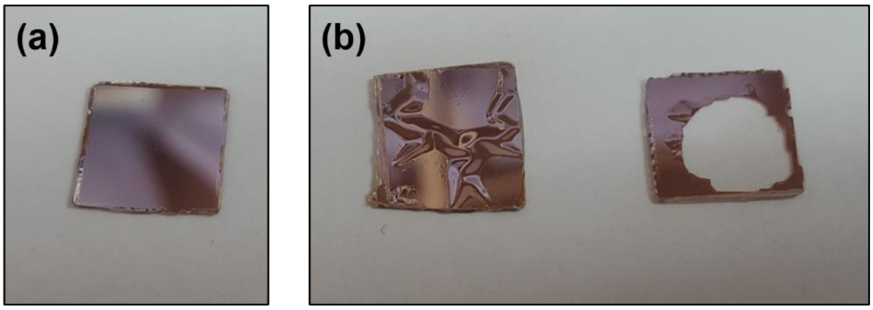

Figure S2. Representative photographs of the red LED thin film transferred onto DSPS when fabricated at (a) optimized curing temperature and (b) above it.

Table S1. Curing and final annealing temperature tests for acquiring optimal bonding.

Curing Temperature	Curing Condition	Annealing Condition after Curing			
		200°C	225°C	250°C	275°C
160°C	O	O	X		
200°C	O	O	O	O	X
250°C	X				

Note. 'O' mark means the sample was optimally transferred without any cracks. While, 'X' mark means the sample was not optimally transferred with cracks.

3. Analysis of the electrical degradation of the transferred red LEDs from SIMS data

The Mg and C acceptor profiles in the *p*-GaP layer measured using SIMS are shown in Figure S3. The *p*-GaP layer of the red LEDs was highly doped with C (~high 10^{19}) from the surface to a depth of 500 nm and relatively less doped with Mg (~low 10^{18}) from a depth of 500 nm to 2 μm. Ohmic-like *p*-metal contact was formed in the Mg doped region due to the inverted structure of the transferred red LEDs, but optimal ohmic contact was formed on the highly doped *p*-GaP surface of the red LEDs on the GaAs wafer. This resulted in better IV characteristics in the red LEDs on the GaAs wafer than the transferred red LEDs. We just used a commercial red LED structure that minor doping tuning in the *p*-GaP layer could improve the IV characteristics in the transferred red LEDs.

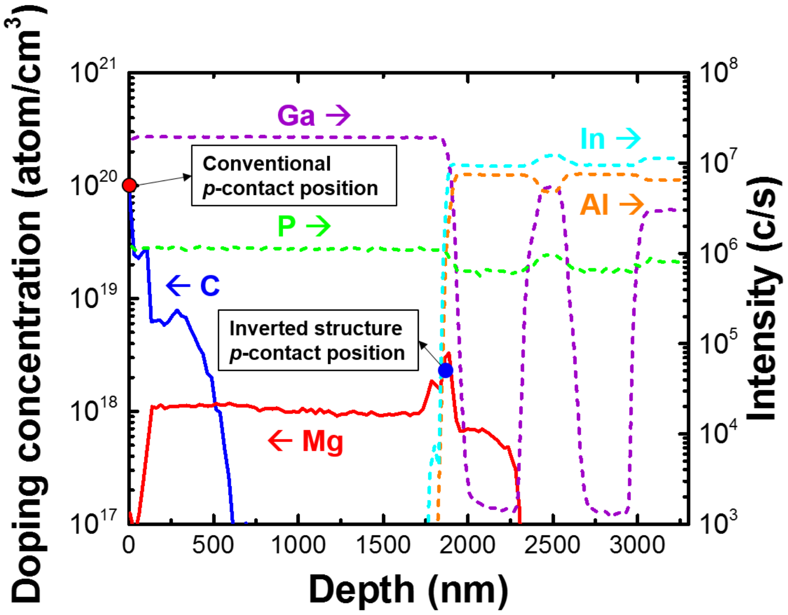

Figure S3. SIMS profiles of Mg and C in the p-GaP layer.

4. Detailed observation of the shoulder peak of the transferred red LEDs and the red LEDs on a wafer

Figure S4 (a) and (b) show the EL spectra of the transferred red LEDs and the red LEDs on a GaAs wafer. The EL spectra of the transferred red LED thin film show a clear shoulder peak (~626 nm) even at a low current level, while a weak shoulder peak can be observed for the red LEDs on a GaAs wafer at a high current level. The increased shoulder peak intensity in the transferred red LED is due to the removal of the GaAs substrate which absorbed light in the ~626 nm wavelength region.

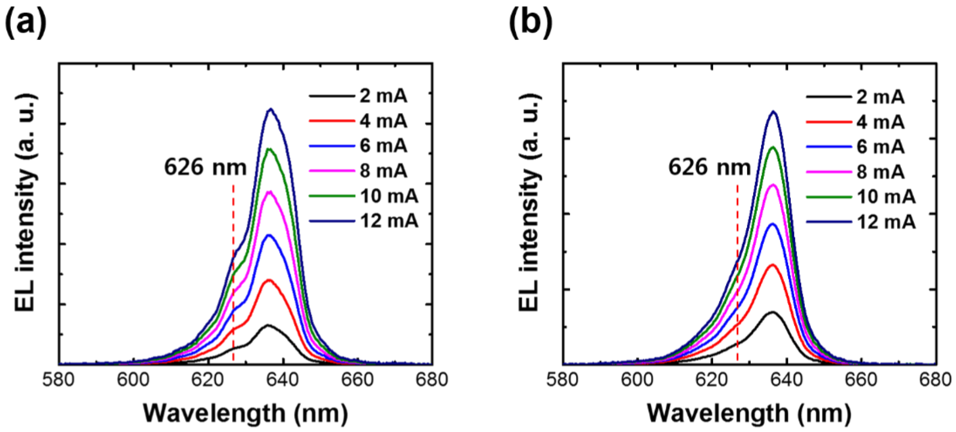

Figure S4. EL spectra of (a) the transferred red LEDs and (b) the red LEDs on wafer. Various current levels from 2 to 12 mA were applied to both.

5. Performance verification of the dual color LEDs having a VSS structure

Figure S5 (a) and (b) shows the I-V characteristics of the red and green subpixels, respectively. For verification of its electrical properties, the VSS-type LED structure was compared to the LAS-type one, in which the electrical properties of the VSS-type LEDs (both red and green subpixels) were found to be similar to those of the LAS-type LEDs. In addition, both structures showed similar results for color control through the optical power ratio (Figure S5 (c) and (d)). This implies that not only the LAS-type structure but also the VSS-type structure can satisfactorily guarantee display device operation and performance.

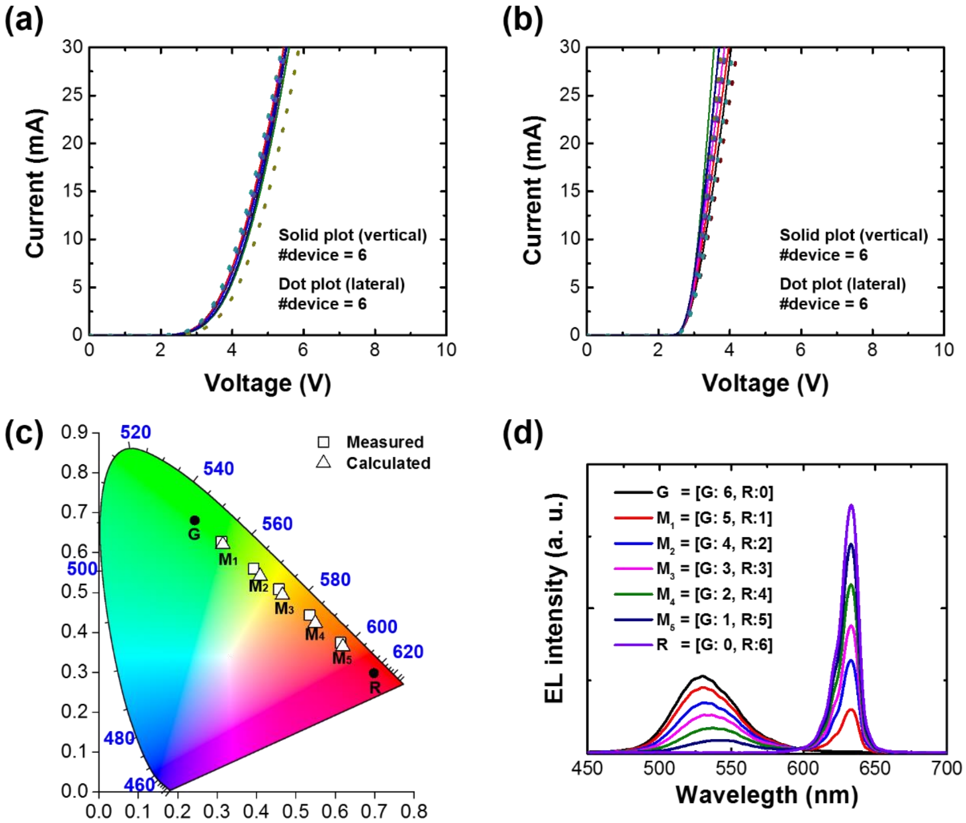

Figure S5. I-V characteristics of (a) red and (b) green subpixels (solid lines: VSS-type structure, dotted lines: LAS-type structure), (c) measured and calculated color coordinates of the dual color LEDs having a VSS-type array structure for the seven color modes (like the LAS-type device), and (d) EL spectra of a VSS-type device measured for the seven color modes.
